# Clinical and genetic features of 334 Asian patients with Birt–Hogg–Dubé syndrome (BHDS) who presented with pulmonary cysts with or without a history of pneumothorax, with special reference to BHDS-associated pneumothorax

**DOI:** 10.1371/journal.pone.0289175

**Published:** 2023-07-25

**Authors:** Yukiko Namba, Hiroki Ebana, Shouichi Okamoto, Etsuko Kobayashi, Masatoshi Kurihara, Yasuhito Sekimoto, Kenji Tsuboshima, Makiko Kunogi Okura, Yoichiro Mitsuishi, Kazuhisa Takahashi, Kuniaki Seyama

**Affiliations:** 1 Division of Respiratory Medicine, Juntendo University Graduate School of Medicine, Tokyo, Japan; 2 The Study Group of Pneumothorax and Cystic Lung Diseases, Tokyo, Japan; 3 Pneumothorax Research Center, Nissan Tamagawa Hospital, Tokyo, Japan; University of Minnesota School of Dentistry, UNITED STATES

## Abstract

**Background:**

The clinical pulmonary manifestations and genetic features of Birt–Hogg–Dubé syndrome (BHDS) in Asian patients remained unclear. We aimed to clarify the clinical features of BHDS-associated pneumothorax (PTX) and retrospectively investigate potential contributing factors in the largest Asian cohort to date.

**Methods:**

We reviewed the clinical and genetic data collected in 2006–2017, from the BHDS patients who were Asian and presented with pulmonary cysts with or without a history of PTX.

**Results:**

Data from 334 (41.3% males; 58.7% females) patients from 297 unrelated families were reviewed. Among them, 314 (94.0%) patients developed PTX. The median age at the first occurrence of PTX was 32 years, which was significantly lower in males (*P* = 0.003) and patients without notable skin manifestations (*P* < 0.001). Seventy-six (24.2%) patients experienced their first PTX episode before the age of 25 years. PTX simultaneously occurred in the bilateral lungs of 37 (11.8%) patients. Among 149 patients who had their first PTX episode at least 10 years before BHDS diagnosis, PTX occurred more frequently in males (*P* = 0.030) and light smokers than in nonsmokers (*P* = 0.014). The occurrence of PTX peaked in the early 30s and gradually decreased with age but remained high in females (*P* = 0.001). We identified 70 unique *FLCN* germline variants, including duplications (46.4%), substitutions (7.1%), insertions/deletions (30.0%), and variants affecting splicing (12.5%). Approximately 80% of Asian patients suspected of having BHDS could be genetically diagnosed by examining *FLCN* exons 7, 9, 11, 12, and 13. No apparent genotype–phenotype correlation regarding pulmonary manifestations was identified.

**Conclusions:**

Our findings indicate that sex, smoking history, and skin manifestations at BHDS diagnosis significantly influence the clinical features of BHDS-associated PTX. These findings may contribute to the appropriate management and treatment of BHDS-associated PTX.

## Introduction

Birt–Hogg–Dubé syndrome (BHDS), also known as Hornstein–Knickenberg syndrome [1], is a rare autosomal dominantly inherited genodermatosis. It is characterized by the presence of fibrofolliculomas on the face, head, and upper torso; pulmonary cysts with or without a history of pneumothorax (PTX); and an increased susceptibility to renal tumors. The identification of *FLCN* in 2001 revealed its role as a causative gene for BHDS [2–4]. *FLCN* encodes a protein known as folliculin [4], which is believed to act as a tumor suppressor, based on Knudson’s two-hit hypothesis, in BHDS-associated renal tumors [5, 6]. However, unlike renal tumors, no somatic variants or loss of heterozygosity has been detected in either skin [7] or pulmonary lesions, suggesting that haploinsufficiency can induce the phenotypes observed in these tissues [8, 9].

BHDS-associated pulmonary manifestations, such as lung cysts and PTX, tend to develop at an earlier age than other organ manifestations, often leading to an early diagnosis of BHDS [10–13]. Pulmonary cysts have been reported in 70%–84% of patients with BHDS [10, 14, 15]. Patients with BHDS have a significantly higher risk of PTX episodes, approximately 50 times higher than their unaffected family members [16], with the first PTX episode frequently occurring before the age of 40 years [12, 13, 17–19]. A previous study reported that among 76 patients with BHDS, 62 (82%) experienced multiple episodes of PTX, with a mean of 3.6 episodes (range = 1 to >8) [13]. However, factors influencing the clinical features of BHDS-associated PTX as well as the presence of genotype–phenotype correlations have remained unclear due to limited sample sizes and the influence of family clustering [20]. This retrospective study aimed to reveal the clinical features of BHDS-associated PTX and investigate potential contributing factors in the largest Asian cohort to date.

## Methods

### Procedures

From 2006 to 2017, we performed *FLCN* genetic testing in patients who were referred to our clinic with a diagnosis of cystic lung disease, with or without a history of PTX. Written informed consent was obtained from all patients for *FLCN* genetic testing. We provided a standardized interview form to referral physicians to collect clinical information from patients suspected of having BHDS. This form was also utilized in our hospital when we encountered and interviewed patients with suspected BHDS. Furthermore, in cases where family members expressed interest in undergoing *FLCN* genetic testing following the diagnosis of BHDS in their proband, we requested referral physicians to collect clinical information using the same standardized interview form.

We retrospectively collected the following information from standardized interview forms at the time of BHDS diagnosis: smoking history, medical history, family history, skin manifestations evaluated by referring pulmonologists (rather than dermatologists), details of PTX episodes (such as number of episodes, age at each occurrence, side of PTX, and simultaneous occurrence in both lungs), and any renal abnormalities. The study protocol was approved by the ethics committee of Juntendo University Hospital (No.2019242). As this study aimed to analyze the clinical and genetic characteristics of BHDS-associated PTX, we included patients who had only been partially reported in previous studies [21–24]. Among 31 patients from the study on BHDS-associated skin manifestations by Iwabuchi et al., we included 28 patients due to the lack of sufficient clinical information regarding PTX and lung cysts [24]. Furthermore, in this study, we decided to incorporate only the assessments of skin manifestations documented by referring pulmonologists rather than those described by dermatologists to maintain consistency in the assessment process. However, discrepancies were noted regarding the presence or absence of skin manifestations in 10 patients [24].

### Analysis of *FLCN* variants

*FLCN* variants were investigated using polymerase chain reaction (PCR) amplification of each exon with primer sets, as described previously [21, 22], followed by direct sequencing. Moreover, a large genomic deletion in *FLCN* was identified by quantifying each *FLCN* exon using real-time quantitative PCR [21]. The nomenclature of the variants was assigned following the recommendations of the Human Genome Variation Society (HGVS) [25]. NM_1449997.7 was considered as the reference sequence for *FLCN*. To determine the relevance of the detected *FLCN* variants to BHDS, we employed the American College of Medical Genetics and Genomics (ACMG) guidelines [26] and searched the ClinVar database (https://www.ncbi.nlm.nih.gov/clinvar/). Some variants were not reported in the ClinVar database; however, as most of them were nonsense or splice site variants, they were classified as pathogenic variants based on the ACMG guidelines. For the variants suspected of affecting splicing, we confirmed their impact on mRNA transcripts through reverse transcription–PCR using total RNA isolated from patients’ peripheral blood mononuclear cells [21]. Further, to predict the impact of the remaining variants, we used Mutation Taster (https://www.mutationtaster.org/), Polyphen-2 (http://genetics.bwh.harvard.edu/pph2/), and our genetic knowledge. Based on the clinical manifestations and characteristic chest CT images of patients with BHDS, we revealed that all detected *FLCN* variants were pathogenic. In the future, we plan to submit the pathogenic variants that have not yet been reported to the ClinVar database.

### Statistical analysis

All statistical analyses were performed using EZR version 1.52 (Saitama Medical Center, Jichi Medical University, Saitama, Japan), a graphical user interface for R version 4.02 (The R Foundation for Statistical Computing, Vienna, Austria) [27]. To assess differences in clinical features, Fisher’s exact test or Student’s t-tests were employed. Factors associated with age at the first occurrence of PTX were analyzed using the Kaplan–Meier method, log-rank test, and Cox proportional hazards models with AIC for variable selection. The frequency of PTX events was assessed using a negative binomial regression model with the offset logarithm function. The distribution of age-related PTX frequency by sex was analyzed using the chi-square test of homogeneity. *P*-values of < 0.05 (two-tailed) were considered to indicate statistical significance.

## Results

### Patient characteristics

Table 1 presents the characteristics of 334 patients with BHDS from 297 unrelated families. Among them, 297 patients were probands, whereas 37 were relatives. The diagnosis of BHDS was confirmed in all patients through the identification of a pathogenic *FLCN* germline variant via *FLCN* genetic testing. Among all patients, 41.3% (N = 138) patients were male, whereas 58.7% (N = 196) were female. The cohort consisted of 328 patients from Japan (98.2%), 5 from China, and 1 from Indonesia. The mean age at BHDS diagnosis was 46.0 ± 14.0 (mean ± SD) years. Approximately 62% (N = 207) of the patients were nonsmokers, whereas 37.1% (N = 124) of them had a history of smoking. Data regarding smoking habits were not available for three patients. No significant differences in background characteristics were noted between the probands and their relatives.

**Table 1:** Characteristics of BHDS patients (N = 334).

		All	Probands	Relatives	*P*
Number of patients	-N (%)	334 (100)	297 (88.9)	37 (11.1)	
Sex*					0.481
Male	-N (%)	138 (41.3)	125 (42.1)	13 (35.1)	
Female	-N (%)	196 (58.7)	172 (57.9)	24 (64.9)	
Age at BHDS diagnosis^‡^					0.385
Mean (± SD)	-yr	46 (± 14.0)	46 (± 13.2)	48 (± 18.9)	
Median (range)	-yr	44 (16–88)	44 (16–85)	45 (16–88)	
Smoking*					0.587
Never smoker	-N (%)	207 (62.0)	181 (61.0)	26 (70.3)	
Ex-smoker	-N (%)	91 (27.2)	83 (27.9)	8 (21.6)	
Current smoker	-N (%)	33 (9.9)	30 (10.1)	3 (8.1)	
Unknown	-N (%)	3 (0.9)	3 (1.0)	0 (0.0)	

Statistically significant differences were determined using the Fisher’s exact test* or student t-tests^‡^.

### Characteristics of patients with BHDS who presented with PTX and/or lung cysts

Table 2 presents the characteristics of 334 patients with BHDS who were categorized into two groups based on their presenting features: “PTX and Cysts” and “Cysts” groups. Among these patients, 314 (94%) patients belonged to the PTX and Cysts group, which was characterized by the occurrence of PTX followed by the detection of multiple lung cysts with distinctive features on chest CT images. The remaining 20 patients (6%) were classified as the Cysts group, in which multiple lung cysts were incidentally identified during chest CT conducted as part of health check-ups or screenings for other diseases. The mean age at BHDS diagnosis was significantly lower in the PTX and Cysts group (46.0 ± 13.8 years) than in the Cysts group (56 ± 14.1 years) (*P* = 0.002). No significant differences were observed between the two groups in terms of sex or smoking history.

**Table 2:** Characteristics of BHDS patients by presenting features (N = 334).

		PTX and Cysts group	Cysts group	*P*
Number of patients	-N (%)	314 (94.0)	20 (6.0)	
Sex*	-N (%)			0.354
Male		132 (33.3)	6 (30.0)	
Female		182 (66.7)	14 (70.0)	
Age at BHDS diagnosis^‡^	-yr			0.002
Mean ± SD		46 (±13.8)	56 (±14.1)	
Median (range)		43 (16–88)	53 (31–76)	
Smoking*	-N (%)			0.250
Never smoker		191 (60.8)	16 (80.0)	
Ex-smoker		88 (28.0)	3 (15.0)	
Current smoker		32 (10.2)	1 (5.0)	
Unknown		3 (1.0)	0 (0)	

Statistically significant differences were determined using the Fisher’s exact test* or student t-tests^‡^.

### Clinical features of the probands at presentation

Table 3 summarizes the clinical features of 297 probands at presentation. All probands had lung cysts, and 277 (93.3%) of them had PTX. Skin manifestations were assessed in 293 probands by referring pulmonologists or our team, and 100 (34.1%) of these patients were found to have papules on their face, neck, or upper chest. Histological examination was not performed for most patients. Among 294 patients with an assessment of renal manifestations, 11 (3.7%) patients had renal tumors and 22 (7.5%) had renal cysts. A history of other neoplasms was detected in 291 probands, with low prevalence at various sites. Family histories of PTX (71.6%), skin manifestations (16.1%), and renal tumors (8%) were noted. Interestingly, 22% of the probands had no family history of lung, skin, or renal manifestations.

**Table 3:** Clinical features of the probands at presentation.

	N (%)
Pulmonary manifestations (N = 297)	
PTX	277 (93.3)
Lung cysts	297 (100.0)
Skin manifestations (N = 293)	
Papules on face, neck, or upper chest*	100 (34.1)
Renal manifestations (N = 294)	
Renal tumors	11 (3.7)
Renal cysts	22 (7.5)
Other clinical tumor manifestations (N = 291)	
Thyroid carcinoma	2 (0.7)
Lung cancer	3 (1.0)
CCST^‡^	1 (0.3)
Gastric cancer	2 (0.7)
GIST	1 (0.3)
Colon cancer	7 (2.4)
Breast cancer	4 (1.4)
Uterine myoma	9 (3.1)
Cervical cancer	2 (0.7)
Endometrial cancer	1 (0.3)
Family history	
PTX (N = 295)	212 (71.6)
Skin lesions (N = 285)	46 (16.1)
Renal tumors (N = 286)	23 (8.0)
No family history (N = 285)	63 (22.0)

The N sizes each differ due to missing patient data.

* Skin manifestations were assessed by referring pulmonologists (not dermatologist) and no histological examination was performed in most patients.

^‡^Clinical features were previously reported [23]. CCST = clear sugar cell tumor (of the lung); GIST = gastrointestinal stromal tumor

### Characteristics of PTX

Table 4 presents the clinical features of PTX in 314 patients who experienced a PTX episode. The mean age at the first occurrence of PTX was 35 ± 13.1 years (N = 312), with a median age of 32 (range = 14–78) years. Among them, 76 (24.2%) patients developed their first PTX episode before the age of 25 years. The right lung was slightly more commonly affected by the first PTX episode than the left lung (51.6% vs. 42.2%). Notably, 19 (6.2%) probands experienced simultaneous bilateral PTX at their initial PTX episode. Further, 77.4% (N = 245) of the patients experienced the recurrence of PTX between their first PTX episode and BHDS diagnosis, with a median duration of 8 (range = 0–50) years. The median number of PTX episodes was 3 (range = 1–15). Simultaneous occurrence of PTX in both lungs was frequently observed in patients with BHDS. A total of 37 patients (11.8%) had bilateral PTX either at their first episode (19 patients) or during the subsequent clinical course (19 patients), and 1 patient experienced simultaneous bilateral PTX both at their first episode and during the subsequent clinical course.

**Table 4:** Characteristics of PTX in BHDS patients (N = 314).

Age at the first PTX episode (N = 312)*	-yr	
	-Mean (± SD)	35 (± 13.1)
	-Median (range)	32.0 (14–78)
First PTX episode at < 25 years of age	-N (%)	76 (24.2)
Recurrence of PTX	-N (%)	245 (77.4)
Number of PTX episodes	-Mean/Median/Range	3.3/3/1–15
Years from first PTX to diagnosis	-Mean/Median/Range	11.1/8/0–50
Side of the first PTX episode (N = 306)^‡^	-N (%)	
Right		158 (51.6)
Left		129 (42.2)
Bilateral (simultaneously)		19 (6.2)
Bilateral PTX episode (simultaneous)		
Number of patients	-N (%)	37 (11.8)^§^
Occurred as the first episode		19 (6.1)
Occurred during the clinical course		19 (6.1)

*Not recalled in 2 patients.

^‡^Not recalled in 8 patients.

^§^1 patient experienced simultaneous bilateral PTX at their first PTX as well as during the subsequent clinical course.

### Factors associated with age at the first PTX episode

**Fig 1:**
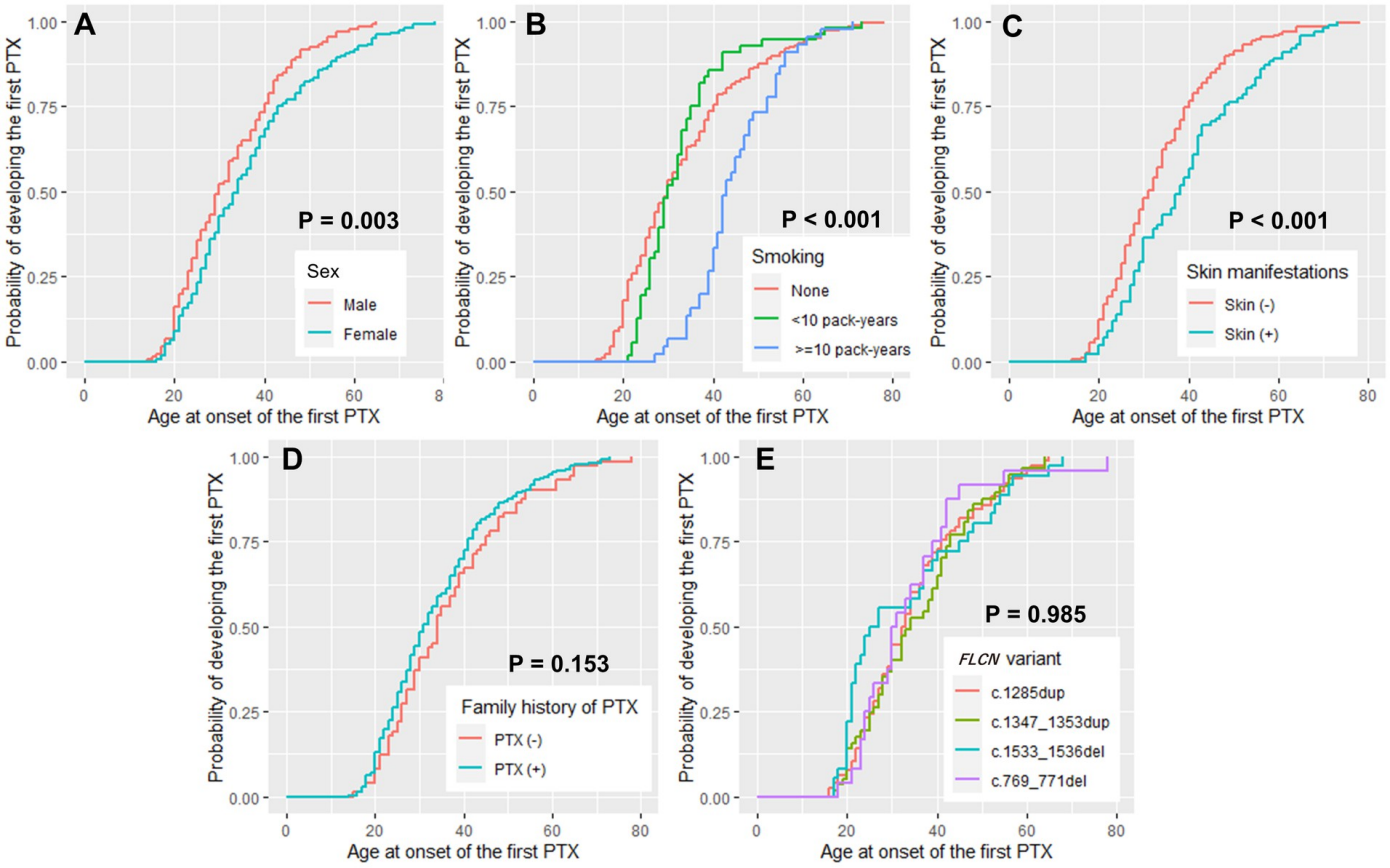
Kaplan–Meier estimates of developing the first PTX (N = 314). The graphs represent (A) sex, (B) smoking history, (C) presence or absence of skin manifestations, (D) family history of PTX, and (E) the 4 common *FLCN* pathogenic variants. The X-axis shows age in years at the first PTX occurrence whereas the probability of developing the first PXT is shown on Y-axis. The *P* values are from the Kaplan–Meier log-rank test.

We investigated the factors potentially associated with age at the first PTX episode in all 314 patients with BHDS who experienced PTX using the Kaplan–Meier method (Fig 1). The Kaplan–Meier log-rank test revealed that males were significantly younger at the first PTX episode than females (*P* = 0.003) (Fig 1A). We further examined the impact of smoking exposure on PTX occurrence by categorizing patients into three groups, namely, nonsmokers, light smokers (<10 pack-years), and heavy smokers (≥10 pack-years), based on the median age at the first occurrence of PTX. The analysis demonstrated a significant association between smoking history and age at the onset of PTX, with heavy smokers experiencing the first PTX episode significantly later than nonsmokers or light smokers (*P* < 0.001) (Fig 1B). Patients with BHDS without notable skin lesions at presentation were significantly younger at the first PTX episode than those with notable skin lesions (*P* < 0.001) (Fig 1C). No significant differences were observed in the age at the first PTX episode based on the family history of PTX (*P* = 0.153) (Fig 1D) or presence of four common *FLCN* pathogenic variants (*P* = 0.985) (Fig 1E).

Furthermore, we conducted both univariate and multivariate analyses using a Cox proportional hazards regression model with AIC model selection to identify the risk factors associated with the early onset of PTX (Table 5). The results revealed significant associations of sex, smoking, and skin manifestations with the onset of the first PTX episode. Males were significantly younger at the first PTX episode than females (hazard ratio, 0.63; 95% confidence interval [CI], 0.45–0.87; *P* = 0.005). Moreover, patients with no notable skin manifestations at presentation were significantly younger at the first PTX episode (hazard ratio, 0.69; 95% CI, 0.50–0.94; *P* = 0.020). Patients with a smoking history of ≥10 pack-years experienced the first PTX episode at a significantly later age than nonsmokers (hazard ratio, 0.47; 95% CI, 0.31–0.71; *P* < 0.001).

**Table 5:** Univariate and multivariate analyses of the age at the first PTX episode (N = 312).

Variable	Age (yr) (median)	Univariate		Multivariate	
		HR (95% CI)	*P*	HR (95% CI)	*P*
Sex (Male vs. Female)	30/34	0.72 (0.57–0.90)	0.004	0.63 (0.45–0.87)	0.005
Family history of PTX (− vs. +)	34/31	1.21 (0.92–1.57)	0.167	1.40 (0.97–2.01)	0.069
Smoking					
None vs. <10 pack-years	30/30	1.12 (0.83–1.51)	0.500	1.38 (0.91–2.10)	0.129
None vs. ≥10 pack-years	30/43	0.56 (0.40–0.77)	<0.001	0.47 (0.31–0.71)	<0.001
Skin manifestations (− vs. +)	31/38	0.66 (0.52–0.85)	<0.001	0.69 (0.50–0.94)	0.020
*FLCN* variant					
c.1285dup vs. c.1347_1353dup	32.5/34	0.95 (0.67–1.33)	0.750	-	-
c.1285dup vs. c.1533_1536del	32.5/26	1.00 (0.67–1.50)	0.986	-	-
c.1285dup vs. c.769_771del	32.5/30.5	1.02 (0.64–1.62)	0.945	-	-

HR = hazard ratio; CI = confidence interval

Statistical analysis utilized the Kaplan–Meier method, log-rank test and Cox proportional hazards models with AIC for selecting variables.

### Factors associated with the frequency of PTX episodes in patients with BHDS

We analyzed the frequency of PTX episodes in patients with BHDS who had a minimum 10-year interval between their first PTX episode and confirmed BHDS diagnosis. Among 314 patients who experienced PTX episodes in this cohort, 149 (47.5%) patients showed the above-mentioned 10-year interval. In this subgroup, we explored various characteristics and potential factors associated with the frequency of PTX episodes per year.

**Fig 2:**
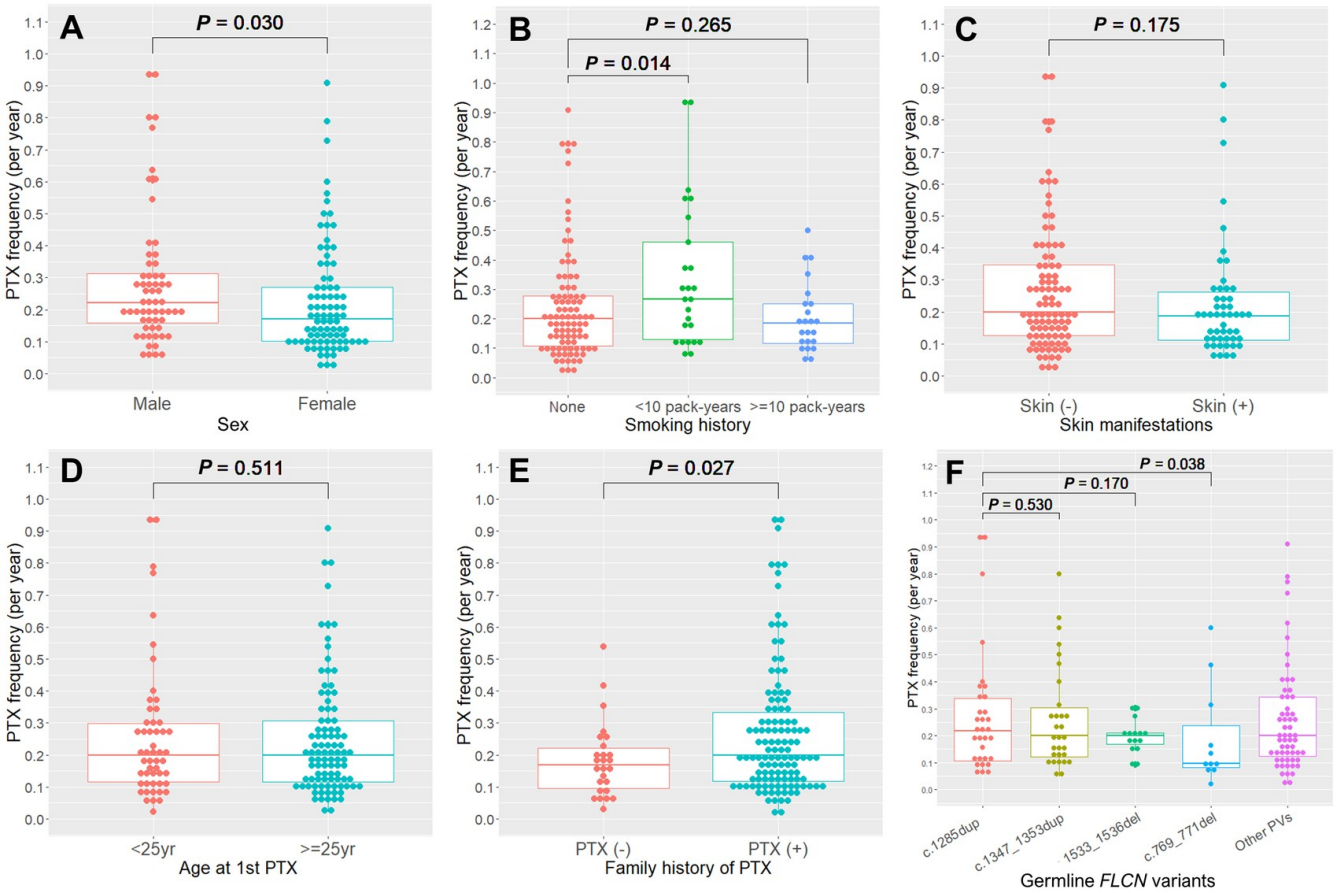
PTX frequency (per year). The PTX frequency per year is shown by (A) sex, (B) smoking history, (C) presence or absence of skin manifestations, (D) the age at first PTX (< 25 vs. ≥ 25 years of age), (E) family history of PTX, and (F) *FLCN* pathogenic variants (the 4 common types). Analyses were performed for patients who had their first PTX ≥10 years before receiving a diagnosis of BHDS (N = 149).

The frequency distribution of PTX episodes in 149 patients with regard to clinical features is presented in Fig 2. The mean frequency of PTX episodes was 0.25 ± 0.19 with a median frequency of 0.20 (range = 0.02–0.93). We conducted both univariate and multivariate analyses using a negative binomial regression model with AIC model selection to identify the risk factors associated with the frequency of PTX episodes (Table 6). In univariate analyses, the frequency of PTX episodes was significantly higher in males than in females (median = 0.22 vs. 0.17; *P* = 0.030) (Fig 2A). Furthermore, the frequency was higher among light smokers than among nonsmokers (median = 0.27 vs. 0.20; *P* = 0.014) (Fig 2B). No statistically significant differences in PTX frequency were noted based on the presence or absence of skin manifestations (Fig 2C) or age at the first PTX occurrence (<25 vs. ≥25 years) (Fig 2D). Patients with a family history of PTX had a significantly higher frequency of PTX episodes than those without any family history (median = 0.20 vs. 0.17; *P* = 0.027) (Fig 2E). In contrast, patients carrying the *FLCN* variant c.769_771del had a significantly lower frequency of PTX episodes than those carrying the variant c.1285dup (median = 0.10 vs. 0.22; *P* = 0.038) (Fig 2F). In the multivariate analysis, only two significant factors were associated with an increased frequency of PTX episodes: male sex (*P* = 0.047) and light smokers (*P* = 0.003). Interestingly, compared with nonsmokers, heavy smokers displayed a trend toward a lower frequency of PTX episodes (*P* = 0.054).

**Table 6:** Univariate and multivariate analyses of PTX frequency per year (N = 147).

Variable	PTX/year		Univariate		Multivariate	
	(average)	(median)				
			Estimate (95% CI)	*P*	Estimate (95% CI)	*P*
Sex (Male vs. Female)	0.29/0.23	0.22/0.17	-0.26 (-0.50 to -0.02)	0.030	-0.32 (-0.63 to -0.01)	0.047
Family history of PTX (− vs. +)	0.18/0.27	0.17/0.20	0.36 (0.04 to 0.68)	0.027	-	-
Smoking						
None vs. <10 pack-years	0.25/0.34	0.20/0.27	0.38 (0.07 to 0.70)	0.014	0.56 (0.18 to 0.93)	0.003
None vs. ≥10 pack-years	0.25/0.20	0.20/0.19	-0.19 (-0.53 to 0.15)	0.265	-0.39 (-0.80 to 0.01)	0.054
Skin manifestations (− vs. +)	0.27/0.23	0.20/0.19	-0.17 (-0.43 to 0.08)	0.175	-0.22 (-0.51 to 0.08)	0.147
Age at 1st PTX (<25yr vs. ≥25yr)	0.26/0.25	0.20/0.20	-0.08 (-0.33 to 0.17)	0.511	0.24 (-0.05 to 0.54)	0.098
*FLCN* variant						
c.1285dup vs. c.1347_1353dup	0.28/0.26	0.22/0.20	-0.11 (-0.45 to 0.24)	0.530	-	-
c.1285dup vs. c.1533_1536del	0.28/0.20	0.22/0.20	-0.30 (0.74 to 0.13)	0.170	-	-
c.1285dup vs. c.769_771del	0.28/0.19	0.22/0.10	-0.51 (-1.01 to -0.01)	0.038	-	-

Statistical analysis utilized a negative binomial regression model with the offset logarithm function.

**Fig 3:**
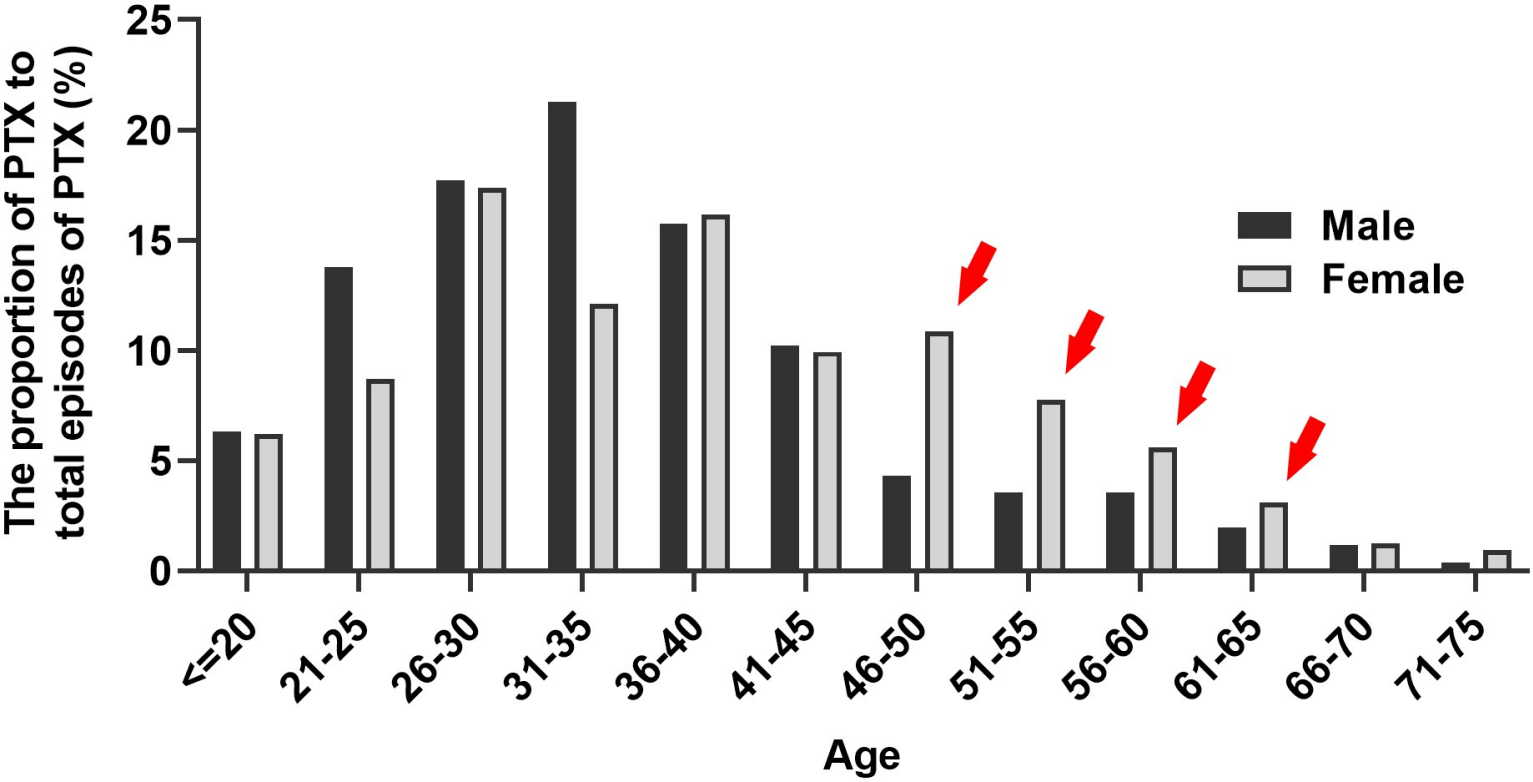
Proportions of PTX episodes for males and females by age (N = 149). The bar graph shows % of total PTX episodes experienced in males and females by age, presented in 5-year increments. Analyses were performed for patients who had their first PTX ≥10 years before receiving a diagnosis of BHDS (N = 149). A total of 322 episodes occurred in 61 males and 254 episodes in 88 females. As indicated by red arrows, females had higher proportions of PTX episodes than males in years 46–65. There was a statistically significant difference in the distribution of age-related PTX frequency between males and females (chi-square test of homogeneity, *P* = 0.001).

We also compared age-related PTX frequency between males and females. Among the total 322 PTX episodes, 61 occurred in males, and 254 occurred in females. Fig 3 presents the proportions of PTX episodes experienced by males and females across different age groups. Ages ranging from ≤20 to 75 years are displayed with 5-year increments. To calculate the proportions, we divided the number of PTX episodes in males and females by the total number of PTX episodes observed in each 5-year period, resulting in 12 age groups. Overall, BHDS-associated PTX appeared to occur most frequently in the age range of 31–35 years, and its frequency gradually decreased with age, although with variations between males and females. In the above-mentioned age range, males showed a single peak of PTX occurrence, whereas females exhibited two peaks. Moreover, females had higher proportions of PTX episodes than males in the age range of 46–65 years. A statistically significant difference in the distribution of age-related PTX frequency was observed between males and females (chi-square test of homogeneity, *P* = 0.001).

### Spectrum of germline *FLCN* pathogenic variants from 297 probands

We identified 70 unique pathogenic *FLCN* variants from 297 probands (S1 Table). These variants consisted of duplications (46.4%), deletions (29.0%), substitutions (7.1%), insertions (0.7%), deletions-insertions (0.3%), large genomic deletions (4.0%), and variants affecting splicing (12.5%). The *FLCN* pathogenic variants were identified in all coding exons and some of their exon/intron boundaries (S2 Table and S1 Fig).

Among the 297 probands, *FLCN* pathogenic variants were detected in 196 (66%) patients across 3 PCR amplicons, namely exons 11 (N = 92), 12 (N = 63), and 13 (N = 41). By including exons 7 (N = 21) and 9 (N = 25), 242 (81.5%) patients could be identified (S2 Table). This finding suggests that the examination of the PCR amplicons encompassing exons 7, 9, 11, 12, and 13 facilitates the genetic diagnosis of BHDS in approximately 80% of Asian patients suspected of having BHDS.

## Discussion

This study included 334 patients with BHDS from 297 unrelated families who presented with pulmonary cysts, with or without a history of PTX. With a focus on analyzing the probands to minimize familial clustering effects, we investigated the association of age at PTX onset and PTX frequency with sex, age, smoking history, and skin manifestations in the largest Asian cohort to date. Among 297 probands, we identified 70 unique germline variants in *FLCN*, distributed across all coding exons and some exon/intron boundaries. No apparent genotype–phenotype correlation was observed regarding the clinical features of BHDS-associated PTX. These findings have potential implications for the management and care of patients with BHDS, particularly those with BHDS-associated PTX, by reducing fears and anxieties of PTX recurrence and improving both patients’ and physicians’ perspectives.

We revealed significant sex differences in the age at first PTX episode and PTX frequency per year. The median age at the first PTX episode was 32.0 (range = 14–78) years and was significantly lower in males than in females. Toro et al. reported that the age at the first PTX occurrence in their cohort (N = 48) was 38 (range 22–71) years, and 88% of them had their first PTX episode before the age of 50 years; moreover, 10% of them had one episode between the ages of 51 and 60 years [17]. In their study involving 197 patients from 67 unrelated families, Sattler et al. reported that the age at the first PTX occurrence was lower in females than in males [20]. In the current study, we observed significant differences in PTX frequency and age-related frequency of PTX between males and females. PTX frequency was higher in males than in females. Additionally, PTX seemed to occur most frequently at ages of 31–35 years, with the frequency gradually decreasing with age in both males and females. Moreover, middle-aged females had a higher proportion of PTX episodes than males.

Additionally, we found that females with BHDS exhibited a second peak of PTX occurrence in the age range of 45–50 years, although the underlying mechanisms remain unknown. This period coincides with the age range at which females undergo menopause, suggesting a potential role of female hormones in PTX recurrence. Notably, a study on primary spontaneous pneumothorax (PSP) reported a lower postoperative recurrence rate in females than in males [28]. We speculate that changes in the levels of female sexual hormones during this period could play a role in the development of the second peak. A previous study reported that the median age at the last PTX episode was 42 (range 22–75) years [17]. These discrepancies may be due to differences in cohort characteristics, such as cohort size, race, sex ratio, and family clustering effects. Our study benefits from a large cohort size (N = 312), with most participants being probands (about 89%), which helps minimize the impact of family clustering effects.

Patients with BHDS-associated PTX exhibit distinct clinical features compared with those with PSP. In PSP, PTX recurrence occurs more frequently in younger individuals (aged approximately 23–25 years) [28, 29]. However, in this BHDS cohort, the frequency of recurrence did not differ between the patients whose first PTX episode occurred earlier than 25 years of age and those who experienced the first episode at or later than 25 years of age. This indicates that the recurrence rate of BHDS-associated PTX is not associated with age, suggesting the existence of different mechanisms of PTX recurrence in patients with BHDS-associated PTX and those with PSP. Additionally, we observed that a high proportion of patients with BHDS (11.8%) experienced simultaneous bilateral PTX. Gupta et al. investigated pulmonary characteristics in 76 patients with BHDS and reported that simultaneous bilateral PTX occurred in 5% of the patients [13]. In PSP, simultaneous bilateral PTX was reported in 1.3%–1.5% of patients, indicating that patients with BHDS have a 7–8 fold higher risk of simultaneous bilateral PTX than those with PSP [30, 31].

We also assessed the influence of smoking history on the onset of BHDS-associated PTX. In heavy smokers, the first PTX episode occurred at a significantly later age than nonsmokers, suggesting that higher smoking levels are not associated with an earlier onset of PTX. However, this result does not indicate a protective effect of smoking on PTX occurrence and could be influenced by bias. Patients who experience PTX are likely to quit smoking subsequently, whereas those who do not have PTX are more likely to continue or start smoking, potentially affecting the observed age at the first PTX episode. Furthermore, PTX frequency was significantly higher among light smokers than among nonsmokers; however, notably, no significant difference in PTX frequency was observed between nonsmokers and heavy smokers.

The impact of smoking on the development of lung cysts and PTX remains unclear. In their study involving 198 patients with BHDS from 89 families, Toro et al. reported that 67% of patients with a history of PTX were nonsmokers and that 61% of patients without a history of PTX episodes were also nonsmokers, suggesting that smoking may not be a significant risk factor for PTX in patients with BHDS [17]. This implies that the effect of smoking on the occurrence of BHDS-associated PTX might be influenced by genetic factors other than *FLCN*. Interestingly, a report indicated that smoking can affect the lung pathology of patients with BHDS. Fabre et al. detected smoking-related respiratory bronchiolitis and subpleural fibroelastotic scars in the lung specimens of one of five patients with BHDS [32]. Furthermore, Li et al. reported that smoking markedly decreased the protein levels of folliculin in the lungs of individuals with chronic obstructive pulmonary disease [33]. They concluded that smoking could promote airway epithelial cell death, which might contribute to the pathogenesis of BHDS-associated PTX. Further studies are warranted to elucidate the precise role of smoking in the development of cysts and PTX in patients with BHDS.

In our cohort, we identified 70 unique *FLCN* germline variants, including four common pathogenic variants (c.1285dup, c.1347_1353dup, c.1533_1536del, and c.769_771del). Upon comparing these frequent pathogenic variants, we did not observe any significant differences in the age at the first occurrence of PTX. However, based on our univariate analysis, patients carrying the c.769_771del variant exhibited a potentially milder phenotype, which may be attributed to their significantly lower PTX frequency, than those carrying the c.1285dup variant.

The pathogenic variant c.1285dup appeared to be associated with a higher incidence of PTX according to a study involving 221 patients from 120 families in China [34]. Additionally, Sattler et al. reported that two pathogenic variants, namely c.1300G>C and c.250−2A>G, were associated with an increased risk of PTX compared with the c.1285dup variant [20]. However, this might be due to the family clustering effect as argued by Hu et al. [35], size of the examined cohort, genetic background factors other than *FLCN*, environmental factors, and other additional factors.

In our study, we only utilized data from probands to minimize the family clustering effect. Additionally, we employed the stepwise variable selection methodology to identify factors associated with age at the first PTX episode or PTX frequency. For age at the first PTX episode, a Cox’s proportional hazards model was utilized, whereas a negative binomial regression model was used for PTX frequency. The procedure for the stepwise variable selection methodology involved systematically adding or removing variables from the model based on predefined criteria, such as *P* value. Initially, we included all variables in the analysis; however, during the stepwise variable selection process, the *FLCN* variant was excluded as it did not contribute significantly to the optimal model. The increased sample size, particularly with regard to each specific variant, may impact the robustness of the multivariate analysis of genotype–phenotype correlation in patients with BHDS-associated PTX. Therefore, further studies with larger cohorts are warranted to provide more comprehensive insights into these relationships.

Our study has several limitations. First, the clinical data of patients were collected retrospectively from the first PTX episode to confirmed BHDS diagnosis. Consequently, it remains unclear whether the analyzed phenotypic traits of BHD-associated PTX persist throughout the patients’ lives. Second, our cohort comprised only patients whose pulmonary manifestations led to the diagnosis of BHDS. This inclusion criterion introduces the potential for ascertainment bias in our cohort. It is important to recognize that different results may have emerged if the cohort included individuals with other phenotypes of BHDS. Nonetheless, our findings still hold significance, considering that the occurrence of PTX and/or detection of multiple pulmonary cysts are typically the earliest indicators prompting the diagnosis of BHDS. Third, the clinical data regarding skin and renal manifestations in our BHDS cohort were not as comprehensive as those regarding pulmonary manifestations. Our primary focus was to elucidate the clinical features of BHDS-associated PTX. Consequently, the assessment of skin and renal manifestations as well as the accuracy of family histories for these manifestations might be less robust. Future studies should aim to collect more comprehensive data on the complete spectrum of BHDS manifestations. Finally, although we did not observe a clear genotype–phenotype correlation regarding pulmonary manifestations, the small sample size for each specific *FLCN* variant could have limited the statistical power of our analysis.

## Conclusions

Based on a cohort of 334 patients with BHDS from 297 unrelated families, this study provides important insights into the clinical features of BHDS-associated PTX along with factors associated with this condition. Our findings contribute to the understanding of the characteristics and expected progression of BHDS-associated PTX. However, considering the limited knowledge regarding the clinical course following the establishment of a BHDS diagnosis, a registry study is warranted to unveil a comprehensive clinical profile of pulmonary manifestations in BHDS. Moreover, it is essential to conduct further investigations to determine whether our findings regarding BHDS-associated PTX in an Asian cohort can be reproduced in individuals from other racial backgrounds. This would help elucidate potential variations in the clinical presentation and outcomes of BHDS-associated PTX across different ethnicities. Comparative studies involving diverse populations may enhance our understanding of the disease and its manifestations.

## Supporting information

S1 Fig The spectrum and distribution of germline *FLCN* pathogenic variants identified in the probands (N = 297) on the genomic structure of *FLCN*. Shown is a schematically depicted genomic structure of *FLCN*, consisting of 14 exons; non-coding exons 1, 2 and 3 are shown with black empty boxes whereas coding exons from 4 to 14 with light blue. The 3’ untranslated region in exon 14 is shown with empty box. *FLCN* pathogenic variants were identified in all coding exons with neighboring exon/intron boundaries. The black bars with shaded ends indicate the large range of genomic deletions with undetermined breakpoints. The numbers after each variant indicate the number of probands who carried either the same variant or type of variant. Among them, *FLCN* pathogenic variants were frequently found in the amplicons including, exon 11 (N = 92, 31.0%), exon 12 (N = 63, 21.2%), exon 13 (N = 41, 13.8%), exon 9 (N = 25, 8.4%), and exon 7 (N = 21, 7.1%) indicating that they were variant hotspots, and collectively accounted for 242 (81.5%) probands. Note that the pathogenic variants ranked in the top 4 highest frequencies are highlighted with blue.
(TIF)

S1 Table Germline *FLCN* pathogenic variants identified and expected alteration of folliculin in the probands (N = 297). To describe *FLCN* germline variants, we used Refseq NM_144997 as the mRNA reference. The nucleotide c.1 corresponds to the adenine of the initiation codon ATG in exon 4.
(DOCX)

S2 Table Locations of germline *FLCN* pathogenic variants identified in the probands (N = 297). * The large genomic deletion in the *FLCN* gene was detected in including exon 1 (N = 1, 0.3%), exons 6–9 (N = 1, 0.3%), exons 9–14 (N = 3, 1.0%), exon 14 (N = 7, 2.4%). ^‡^ 242 (81.5%) of the probands’ germline *FLCN* pathogenic variants were located in amplicon exons 7, 9, 11, 12, and 13.
(DOCX)

S1 File Data set. (XLSX)
