# Supplementary figures and images for: Clinical and genetic features of 334 Asian patients with Birt–Hogg–Dubé syndrome (BHDS) who presented with pulmonary cysts with or without a history of pneumothorax, with special reference to BHDS-associated pneumothorax
Source: PLoS One. 2023 Jul 25;18(7):e0289175. doi: 10.1371/journal.pone.0289175 (PMC10368292; doi:10.1371/journal.pone.0289175)

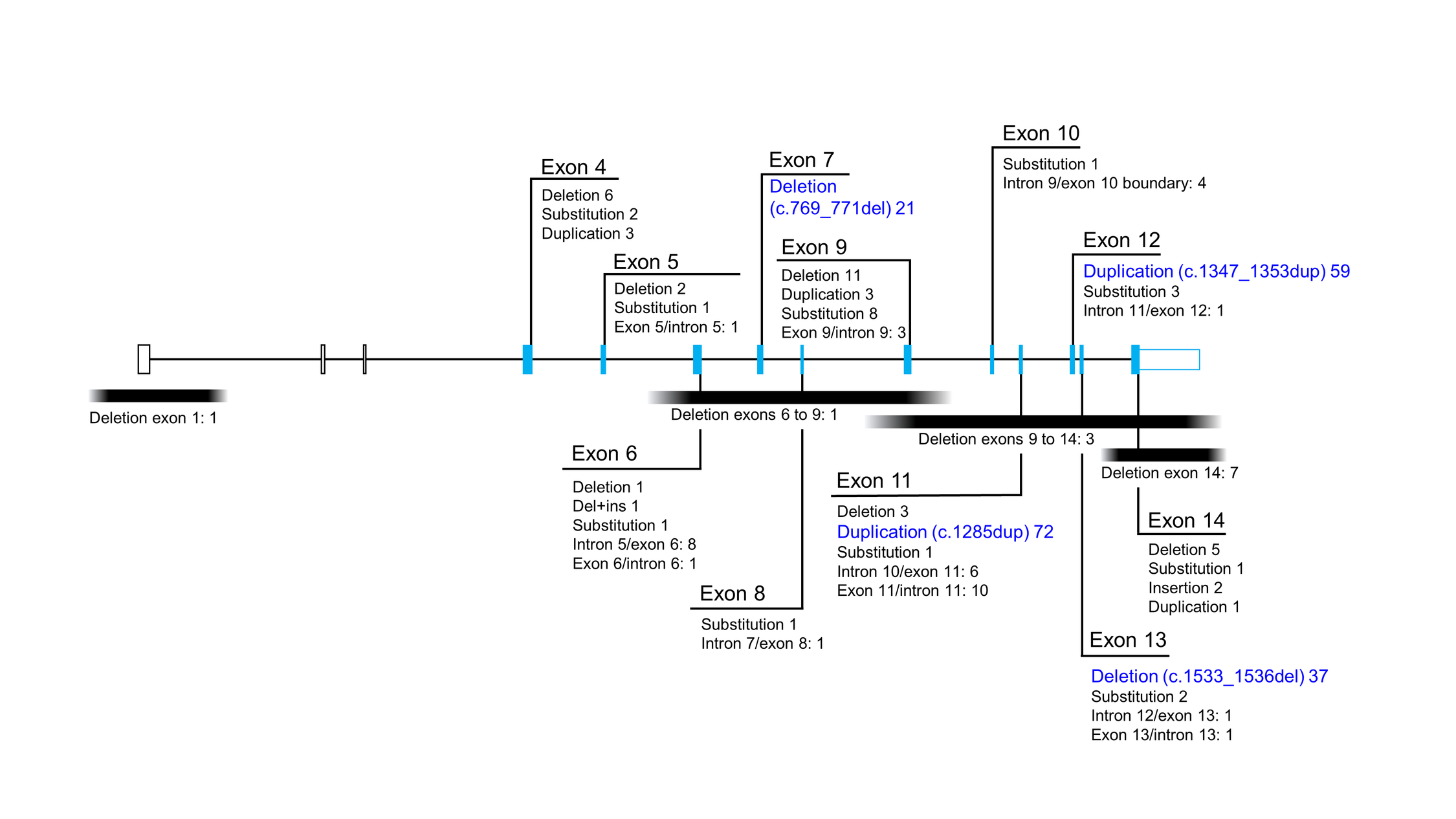

Supplement: S1 Fig — Shown is a schematically depicted genomic structure of FLCN, consisting of 14 exons; non-coding exons 1, 2 and 3 are shown with black empty boxes whereas coding exons from 4 to 14 with light blue. The 3’ untranslated region in exon 14 is shown with empty box. FLCN pathogenic variants were identified in all coding exons with neighboring exon/intron boundaries. The black bars with shaded ends indicate the large range of genomic deletions with undetermined breakpoints. The numbers after each variant indicate the number of probands who carried either the same variant or type of variant. Among them, FLCN pathogenic variants were frequently found in the amplicons including, exon 11 (N = 92, 31.0%), exon 12 (N = 63, 21.2%), exon 13 (N = 41, 13.8%), exon 9 (N = 25, 8.4%), and exon 7 (N = 21, 7.1%) indicating that they were variant hotspots, and collectively accounted for 242 (81.5%) probands. Note that the pathogenic variants ranked in the top 4 highest frequencies are highlighted with blue. (TIF) [file pone.0289175.s001.tif]
