# Supplementary material for: Clinical and genetic features of 334 Asian patients with Birt–Hogg–Dubé syndrome (BHDS) who presented with pulmonary cysts with or without a history of pneumothorax, with special reference to BHDS-associated pneumothorax
Source: PLoS One. 2023 Jul 25;18(7):e0289175. doi: 10.1371/journal.pone.0289175 (PMC10368292; doi:10.1371/journal.pone.0289175)
Supplement: S1 Table — To describe FLCN germline variants, we used Refseq NM_144997 as the mRNA reference. The nucleotide c.1 corresponds to the adenine of the initiation codon ATG in exon 4. (DOCX) [file pone.0289175.s002.docx]

**S1 Table. Germline *FLCN* pathogenic variants identified and expected alteration of folliculin in the probands (N = 297)**

| No. of Unrelated Families Identified | Location | *FLCN* Germline Variant  (NM_144997.7) | Type of Variant | Expected Alteration of Folliculin  (NM_144997.7) |
| --- | --- | --- | --- | --- |
| 1 | Exon 4 | c.57_58 del | Deletion | p.(Phe20Leufs*16) |
| 1 | Exon 4 | c.65C>A | Substitution | p.(Thr22Lys) |
| 2 | Exon 4 | c.119del | Deletion | p.(Gly40Alafs*15) |
| 1 | Exon 4 | c.156dup | Duplication | p.(Gln53Serfs*47) |
| 2 | Exon 4 | c.168_193del | Deletion | p.(Ser56Argfs*35) |
| 1 | Exon 4 | c.179dup | Duplication | p.(His61Alafs*39) |
| 1 | Exon 4 | c.185del | Deletion | p.(Ser62Thrfs*68) |
| 1 | Exon 4 | c.199dup | Duplication | p.(Ala67Glyfs*33) |
| 1 | Exon 4 | c.236C>A | Substitution | p.(Ser79*) |
| 1 | Exon 5 | c.328C>T | Substitution | p.(Gln110*) |
| 2 | Exon 5 | c.332_349del | Deletion | p.(His111_Gln116del) |
| 1 | Exon 5/intron 5 boundary | c.396+1G>A | Splicing |  |
| 1 | Intron 5/exon 6 boundary | c.397-13_397-4del | Splicing |  |
| 1 | Intron 5/exon 6 boundary | c.397-2A>C | Splicing |  |
| 1 | Intron 5/exon 6 boundary | c.397-1G>T | Splicing |  |
| 5 | Intron 5/exon 6 boundary | c.397-7_399del | Splicing |  |
| 1 | Exon 6 | c.402del | Deletion | p.(Pro135Leufs*42) |
| 1 | Exon 6 | c.566_577delinsCC | Deletion-insertion | p.(Leu189Profs*31) |
| 1 | Exon 6 | c.601C>T | Substitution | p.(Gln201*) |
| 1 | Exon 6/intron 6 boundary | c.618+1G>A | Splicing |  |
| 21 | Exon 7 | c.770_772del | Deletion | p.(Ser257del) |
| 1 | Intron 7/exon 8 boundary | c.780-2A>G | Splicing |  |
| 1 | Exon 8 | c.853C>T | Substitution | p.(Gln285*) |
| 1 | Exon 9 | c.887C>A | Substitution | p.(Ser296*) |
| 2 | Exon 9 | c.889_890del | Deletion | p.(Glu297Lysfs*6) |
| 1 | Exon 9 | c.890_893del | Deletion | p.(Glu297Alafs*25) |
| 2 | Exon 9 | c.906dup | Duplication | p.(Glu303*) |
| 1 | Exon 9 | c.907G>T | Substitution | p.(Glu303*) |
| 1 | Exon 9 | c.922G>T | Substitution | p.(Glu308*) |
| 1 | Exon 9 | c.922del | Deletion | p.(Glu308Argfs*15) |
| 2 | Exon 9 | c.932_933del | Deletion | p.(Pro311Argfs*78) |
| 2 | Exon 9 | c.946_947del | Deletion | p.(Ser316Tyrfs*73) |
| 1 | Exon 9 | c.989_990C[4] | Duplication | p.(Ser331Profs*23) |
| 2 | Exon 9 | c.997_998del | Deletion | p.(Ser333Argfs*56) |
| 2 | Exon 9 | c.998C>G | Substitution | p.(Ser333*) |
| 1 | Exon 9 | c.1007_1010del | Deletion | p.(Gly336Alafs*16) |
| 2 | Exon 9 | c.1015C>T | Substitution | p.(Gln339*) |
| 1 | Exon 9 | c.1060C>T | Substitution | p.(Gln354*) |
| 3 | Exon 9/intron 9 boundary | c.1062+1G>A | Splicing |  |
| 1 | Intron 9/exon 10 boundary | c.1063-2A>G | Splicing |  |
| 3 | Intron 9/exon 10 boundary | c.1063-10_1065del | Splicing |  |
| 1 | Exon 10 | c.1126T>A | Substitution | p.(Trp376Arg) |
| 6 | Intron 10/exon 11 boundary | c.1177-5_1177-3del | Splicing |  |
| 1 | Exon 11 | c.1252del | Deletion | p.(Leu418Trpfs*50) |
| 1 | Exon 11 | c.1273C>T | Substitution | p.(Gln425*) |
| 2 | Exon 11 | c.1285delC | Deletion | p.(His429Thrfs*39) |
| 72 | Exon 11 | c.1285dup | Duplication | p.(His429Profs*27) |
| 6 | Exon 11/intron 11 boundary | c.1300+1G>A | Splicing |  |
| 4 | Exon 11/intron 11 boundary | c.1300+2T>C | Splicing |  |
| 1 | Intron 11/exon 12 boundary | c.1301-2A>G | Splicing |  |
| 59 | Exon 12 | c.1347_1353dup | Duplication | p.(Val452Profs*6) |
| 1 | Exon 12 | c.1390G>T | Substitution | p.(Glu464*) |
| 2 | Exon 12 | c.1429C>T | Substitution | p.(Arg477*) |
| 1 | Intron 12/exon 13 boundary | c.1433-1G>T | Splicing |  |
| 1 | Exon 13 | c.1489_1490del | Deletion | p.(Val497Glyfs*23) |
| 1 | Exon 13 | c.1511_1512del | Deletion | p.(Leu504Argfs*16) |
| 1 | Exon 13 | c.1522_1524del | Deletion | p.(Lys508del) |
| 34 | Exon 13 | c.1533_1536del | Deletion | p.(Trp511*) |
| 2 | Exon 13 | c.1533G>A | Substitution | p.(Trp511*) |
| 1 | Exon 13/intron 13 boundary | c.1538+1G>T | Splicing |  |
| 1 | Exon 14 | c.1553T>C | Substitution | p.(Leu518Pro) |
| 1 | Exon 14 | c.1579_1580insA | Insertion | p.(Arg527Glnfs*75) |
| 1 | Exon 14 | c.1589dup | Duplication | p.(Asp531Glyfs*71) |
| 1 | Exon 14 | c.1597_1598del | Deletion | p.(Gln533Glufs*68) |
| 4 | Exon 14 | c.1599_1600del | Deletion | p.(Lys534Alafs*67) |
| 1 | Exon 14 | c.1665_1666ins[TCA;1630_1653;GTCT] | Insertion | p.(Gly556Serfs*56) |
| 1 | Exon 1 | c.1-455-?_c.1-228+?del | Large Deletion |  |
| 1 | Exon 6-9 | c.397-?_c.1062+?del | Large Deletion |  |
| 3 | Exon 9-14 | c.872-?_c.1740+?del | Large Deletion |  |
| 7 | Exon 14 | c.1539-?_c.1740+?del | Large Deletion |  |

To describe *FLCN* germline variants, we used Refseq NM_144997.7 as the mRNA reference. The nucleotide c.1 corresponds to the adenine of the initiation codon ATG in exon 4.
