# Supplementary material for: Clinical and genetic features of 334 Asian patients with Birt–Hogg–Dubé syndrome (BHDS) who presented with pulmonary cysts with or without a history of pneumothorax, with special reference to BHDS-associated pneumothorax
Source: PLoS One. 2023 Jul 25;18(7):e0289175. doi: 10.1371/journal.pone.0289175 (PMC10368292; doi:10.1371/journal.pone.0289175)
Supplement: S2 Table — * The large genomic deletion in the FLCN gene was detected in including exon 1 (N = 1, 0.3%), exons 6–9 (N = 1, 0.3%), exons 9–14 (N = 3, 1.0%), exon 14 (N = 7, 2.4%). ‡ 242 (81.5%) of the probands’ germline FLCN pathogenic variants were located in amplicon exons 7, 9, 11, 12, and 13. (DOCX) [file pone.0289175.s003.docx]

**S2 Table. Locations of germline *FLCN* pathogenic variants identified in the probands (N = 297)**

| Location of *FLCN* | N (%) |
| --- | --- |
| Amplicon exon 4 | 11 (3.7) |
| Amplicon exon 5 | 4 (1.3) |
| Amplicon exon 6 | 12 (4.0) |
| Amplicon exon 7 | 21 (7.1) |
| Amplicon exon 8 | 2 (0.7) |
| Amplicon exon 9 | 25 (8.4) |
| Amplicon exon 10 | 5 (1.7) |
| Amplicon exon 11 | 92 (31.0) |
| Amplicon exon 12 | 63 (21.2) |
| Amplicon exon 13 | 41 (13.8) |
| Amplicon exon 14 | 9 (3.0) |
| Large genomic deletion* | 12 (4.0) |
| Total | 297 (100) |
| Amplicon exons 7, 9, 11, 12, and 13^‡^ | 242 (81.5) |

* The large genomic deletion in the *FLCN* gene was detected in including exon 1 (N = 1, 0.3%), exons 6 – 9 (N = 1, 0.3%), exons 9 – 14 (N = 3, 1.0%), exon 14 (N = 7, 2.4%).

^‡^ 242 (81.5%) of the probands' germline *FLCN* pathogenic variants were located in amplicon exons 7, 9, 11, 12, and 13.
